# Mesenchymal stem cells inhibit multiple myeloma cells via the Fas/Fas ligand pathway

**DOI:** 10.1186/scrt322

**Published:** 2013-09-11

**Authors:** Ikiru Atsuta, Shiyu Liu, Yasuo Miura, Kentaro Akiyama, Chider Chen, Ying An, Songtao Shi, Fa-Ming Chen

**Affiliations:** 1 Center for Craniofacial Molecular Biology, University of Southern California School of Dentistry, Los Angeles, CA 90033, USA; 2 Section of Removable Prosthodontics, Division of Oral Rehabilitation, Faculty of Dental Science Kyushu University, Fukuoka 812-8582, Japan; 3 Translational Research Team, School of Stomatology, Fourth Military Medical University, Xi’an 710032, Shaanxi, P.R. China; 4 Department of Hematology and Laboratory Medicine, Osaka Red Cross Hospital, Osaka 543-8555, Japan; 5 Current address: Department of Oral Rehabilitation and Regenerative Medicine, Okayama University Graduate School of Medicine, Dentistry and Pharmaceutical Sciences, 2-5-1 Shikata-cho, Okayama, Kita-ku 700-8525, Japan; 6 Department of Periodontology and Oral Medicine, School of Stomatology, Fourth Military Medical University, Xi’an 710032, Shaanxi, P.R. China

## Abstract

**Introduction:**

Cell-based therapy represents a new frontier in the treatment of a wide variety of human diseases traditionally associated with morbidity outcomes, including those involving inflammation, autoimmunity, tissue damage, and cancer. However, the use of mesenchymal stem cells (MSCs) to treat multiple myeloma (MM) bone disease has raised concerns. Specifically, evidence has shown that infused MSCs might support tumor growth and metastasis.

**Methods:**

In this study, we used a standard disseminated MM model in mice to identify the *in vivo* effects of intravenous MSC infusion. In addition, a series of *in vitro* co-culture assays were preformed to explore whether Fas/Fas ligand (Fas-L) is involved in the inhibitory effects of MSCs on MM cells.

**Results:**

In the MM mouse model, treatment of MSCs with highly expressed Fas ligand (Fas-L^high^ MSCs) showed remarkable inhibitory effects on MM indenization in terms of extending the mouse survival rate and inhibiting tumor growth, bone resorption in the lumbus and collum femoris, and MM cell metastasis in the lungs and kidneys. In addition, reduced proliferation and increased apoptosis of MM cells was observed when co-cultured with Fas-L^high^ MSCs *in vitro*. Furthermore, mechanistically, the binding between Fas and Fas-L significantly induced apoptosis in MM cells, as evidenced through an increase in the expression of apoptosis marker and Fas in MM cells. In contrast, Fas-L^null^ MSCs promote MM growth.

**Conclusions:**

These data suggest that Fas/Fas-L-induced MM apoptosis plays a crucial role in the MSC-based inhibition of MM growth. Although whether MSCs inhibit or promote cancer growth remains controversial, the levels of Fas-L expression in MSCs determine, at least partially, the effects of MSCs on MM cell growth.

## Introduction

Multiple myeloma (MM) is the second most common hematological malignancy, with a yearly incidence of 14,000 in the United States, constituting 13% of blood cancers and 1% of all human cancers [1]. MM is unique among most hematological malignancies, with a high capacity to induce osteolytic bone lesions through the suppression of osteoblastogenesis, stimulation of osteoclastogenesis and the subsequent uncoupling of bone resorption and bone formation in areas adjacent to tumor foci in the bone marrow [2]. The occurrence of bone lesions is thus one of the major characteristics of MM patients [3]; the number of osteolytic or focal lesions has been associated with clinical disease progression for patients with MM [4]. More than 80% of MM patients suffer from large osteolytic lesions, and a lack of clinically effective therapeutics leads to the increasing severity of skeletal complications and, indeed, death as a result of lowered resistance to infection (that is, immunodeficiency), hypercalcemia, anemia, and renal failure, among others [5]. It is estimated that MM, with a median overall survival of 3 to 5 years, accounts for approximately 20% of deaths from hematologic malignancy and 1 to 2% of cancer-related deaths overall [6]. Currently, cure is a realistic goal for only a small minority of MM patients. The high morbidity and mortality rates associated with plasma cell malignancy have led to an increased demand for the effective management of this condition.

It is now generally accepted that MM bone disease is a reflection of osteoblast deactivation [3,7]. The correction of osteoblast function in the bone of MM patients, therefore, has long been a primary target for the design of therapeutics for MM-related bone disease. To this end, bisphosphonates, such as pamidronate, and novel agents, such as lenalidomide, dasatinib, and bortezomib, which inhibit osteoclast activity and/or activate osteoblasts, have been identified for treatment of MM-induced osteoblast deactivation [2,8]. Increased knowledge of the signaling pathways involved in the regulation of osteoblast formation and differentiation from MSCs might provide a better understanding of the pathophysiological mechanisms involved in MM-induced osteoblast inhibition and permit the development of more potential therapeutic agents against bone damage [2,8,9]. In this regard, novel osteoblast-activating agents, such as dickkopf-1-neutralizing antibody [10] and inhibitor of activin A signaling [11], are explored for the clinical treatment of MM bone disease. The combination of traditional cytotoxic and novel agents has led to higher response rates and improved long-term survival compared with treatment with standard doses of chemotherapy alone. Unfortunately, not all patients will respond to established novel agents, and even those who do respond will ultimately relapse or become refractory to currently available regimens. Moreover, large lytic lesions are typically not repaired, even after long-term remission, and relapses often occur in pre-existing lesions [4]. Consequently, additional approaches are urgently needed to achieve systemic bone anabolism and repair large osteolytic lesions.

The use of mesenchymal stem cells (MSCs), also called mesenchymal stem cells, to treat MM bone disease has received considerable attention in the field of stem cell research [12]. However, whether MSC infusion inhibits or promotes cancer growth remains controversial. A number of *in vitro* studies suggest that MSCs from MM patients possess abnormal genomic, phenotypic, and functional properties, which might contribute to impaired bone formation in this disease by supporting and protecting MM cells from spontaneous and drug-induced apoptosis [9]. Furthermore, recent evidence shows that MSCs, when injected subcutaneously, promote tumor growth and neovascularization in syngeneic mouse models through directly supporting the tumor vasculature and secreting proangiogenic factors [13]. Indeed, the promotion of tumor growth through MSCs has also been observed in various cancer models (reviewed in [14]), suggesting that, at least in some specific conditions, MSCs play important roles in tumor progression. In contrast with evidence supporting the fact that MSCs stimulate tumor growth, other studies have documented the routine suppression of tumor growth through MSCs (also reviewed in [14]). In particular, exogenously administered MSCs effectively promote bone formation and inhibit bone disease and the growth of highly aggressive MM cells in the bone, although the majority of systemically injected MSCs were localized in the lungs or in draining lymph nodes [15]. Furthermore, intrabone-injected MSCs have been demonstrated to act as bystander cells to promote bone formation, inhibit osteolysis, and delay MM growth and regrowth [5,15]. New insights into the effects of *in vivo* milieu on MSC functions might explain these contradicting results [16,17]. Notably, a high dose of melphalan with autologous stem cell support has played an integral part in MM therapy for more than 25 years, either as salvage therapy or to consolidate initial remission, although these therapeutic regimens typically utilize MM cells as adjuvants for other therapeutic agents [12]. Moreover, after MSC transplantation in over 1,000 patients with a clinically acceptable safety profile, not a single case of MSC-related tumors has been reported in a variety of indications [14]. Conceptually, it is a small leap from the adjuvant use of stem cells to novel cell-based therapies to enhance the therapeutic outcome of MM, but the idea has only recently begun to gain momentum.

The clinical and molecular characteristics of MM-related osteolytic lesions support the potential success of cell-based therapies for this disease [5,12,15], where the exogenous administration of healthy MSCs might affect MM bone disease via the secretion of trophic factors, instead of, or in addition to, directly participating in the regeneration of the damaged bone [12]. Gunn and colleagues showed that an interaction between MM cells and MSCs from the bone marrow stroma stimulated the production of dickkopf-1 and IL-6, resulting in the formation and persistence of osteolytic bone lesions [18]. These authors also showed that the Wnt signaling activator 6-bromoindirubin-3′-monoxime might release MSCs from the osteoinhibitory effects of Dickkopf-1, enabling released MSCs to repair existing osteolytic lesions [18]. Following the adjuvant use of stem cells for MM therapy [12], Li and colleagues proposed a proof-of-concept that healthy MSCs, independent of other therapeutic agents, might attenuate the growth of MM and suppress MM-induced bone disease through the inhibition of osteoclastogenesis and stimulation of endogenous osteoblastogenesis [5,15]. Taken together, these data lead to new insights into, and the further exploration of, stem cell-based therapeutics for MM patients.

In addition to altering the bone marrow milieu that favors MM cell accommodation, the therapeutic effects of exogenously infused MSCs might also root from healthy MSC-induced MM cell death/apoptosis [5]. However, the underlying crosstalk between MSCs and MM cells *in vitro* and *in vivo* remains unknown. The execution of programmed cell death is a process triggered through many factors, such as radiation, chemotherapeutic drugs, and apoptotic signaling, which occurs via intrinsic and extrinsic pathways. Both pathways stimulate an intracellular cascade of events leading to cell death. The intrinsic pathway is initiated by mitochondria, whereas the extrinsic pathway is activated through death receptors that engage their respective ligands on the surface membrane of target cells. Fas (DR2/CD95/Apo-1) is a type I cell membrane protein with an extracellular domain that binds Fas ligand (Fas-L) and a cytoplasmic domain that transduces the death signal [19,20]. Fas-L (CD95L/CD178/Apo-1 L) is a type II cell membrane protein belonging to the TNF family, which is inducibly expressed in lymphocytes and constitutively expressed in cells present in immune-privileged organs [21,22]. Fas-L interacts with its receptor, Fas, triggering a cascade of subcellular events culminating in apoptotic cell death [23]. Although Fas/Fas-L interactions play an important role in inducing cell apoptosis, it remains unclear whether Fas/Fas-L is involved in the inhibitory effects of exogenously infused MSCs on MM cells. The purpose of the present study was therefore to determine whether MSCs exert apoptosis-inducing effects on MM cells *in vitro* and *in vivo* through altering Fas/Fas-L expression.

## Materials and methods

### Multiple myeloma cell line

The 5TGM1 MM cell line used in the present study was subcloned from a stroma-independent cell line originally established from parent murine 5 T33 (IgG_2b_κ) MMs [24] and grown in long-term suspension culture in Isocove’s modified Dulbecco’s medium (Invitrogen Co., Carlsbad, CA, USA) with 10% fetal bovine serum (Summit Biotechnology, Fort Collins, CO, USA) and antibiotics. In the tracing experiment, MM cells were stained with carboxyfluorescein diacetate, succinimidyl ester (Invitrogen Co.).

### Isolation of bone marrow mesenchymal stem cells

Bone marrow cells were flushed from the bone cavity of femurs and tibias of B6 mice (C57BL/6 J) with 2% heat-inactivated fetal bovine serum (Equitech-Bio, Kerrville, TX, USA) in PBS, and MSCs positive for CD73, CD90, CD105, CD146, CD166, Sca-1 and SSEA-4, but negative for CD11b, CD31, CD34 and CD45, were obtained as previously described [25]. The cells with MSC character were cultured with alpha minimum essential medium (Invitrogen) supplemented with 20% fetal bovine serum and 2 mM l-glutamine (Invitrogen) in a humidified atmosphere of 95% air and 5% CO_2_ at 37°C for 3 days before co-culture [26]. Additionally, MSCs, which had no Fas-L, were isolated from generalized lymphoproliferative disease (gld) mice for use in comparative experiments. B6 and gld mice in the same background (B6Smn.C3-Faslgld/J) were purchased from the Jackson Laboratory.

### 5TGM1 multiple myeloma model and MSC administration

**Figure 1:**
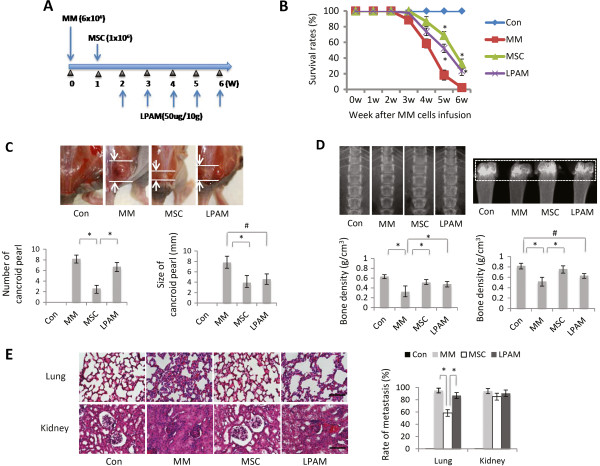
**Effect of mesenchymal stem cell infusion in multiple myeloma model mice. (A)** Experimental protocol of multiple myeloma (MM) cell and mesenchymal stem cell (MSC) injection and the design of lymphocyte Peyer’s patch adhesion molecule (LPAM) administration. **(B)** Six-week survival rates of animals in different groups (for each group, *n* = 14). The survival rate in the nontreated MM model group was dramatically decreased after 3 weeks. The MSC and L-PAM groups had almost the same survival rate, but were higher than the MM group (*P* <0.05). Data shown as the mean ± standard deviation (SD) of three parallel experiments. *P <0.05 versus MM group. **(C)** Comparison of clinical findings in each group. The number (left) and size (right) of cancroid pearls in the neck, root of tail and abdominal cavity are shown as column graphs (top panel). Data shown as mean ± SD for three parallel experiments. *P <0.05 versus MSC group; #*P* <0.05 versus L-PAM group. Lower panel: representative photograph of typical cancroid pearls in the base of the tail (bar = 5 mm). **(D)** Comparison of bone resorption in lumbus and collum femoris in each group. Radiographs of lumbus and collum femoris at 3 weeks after MM cell injection, and the bone density in each group was analyzed. Data shown as mean ± SD for four parallel experiments. *P <0.05 versus MM group; #*P* <0.05 versus L-PAM group. **(E)** Myeloma cell metastasis in the lungs and kidneys in each group. Data shown as mean ± SD for three parallel experiments. *P <0.05 versus MSC group. Left panels: light micrographs of the lungs and kidneys (hematoxylin and eosin staining, bar = 100 μm). Con, control.

A 5TGM1 MM model was conducted in weight-matched, 8-week-old to 10-week-old female bg-nu/nu-xid mice from the Jackson Lab (Bar Harbor, ME, USA). The use of animals for research was approved through the Institutional Ethics Committee/Institutional Review Board of the University of Southern California (protocol #10941). The mice were housed in isolator cages, and autoclaved chow and acidified water were provided *ad libitum*. Disseminated MM was induced through the intravenous inoculation of 6 × 10^6^ 5TGM1 cells in 200 μl PBS in bg-nu/nu-xid mice through the tail vein (6 × 10^6^/10 g body weight). After tumor cell inoculation, multiple MM model mice were randomized to receive the injection of either MSCs (once, 1 × 10^6^ MSCs/10 g body weight) via the tail vein (MSC group) or lymphocyte Peyer’s patch adhesion molecules (L-PAM; weekly, 50 μg/10 g body weight) in the abdominal cavity (L-PAM group) (Figure 1A) [27]. l-Phenylalanine mustard, or L-PAM, otherwise known as melphalan, is used as the standard treatment in older MM patients [28]. MM model mice or original nude mice without any treatments served as positive controls (MM group) or negative controls (Control group). The 6-week survival rates of the mice in different groups were compared. After 4 weeks of feeding, the cancroid pearls in the neck, tail root and abdominal cavity of the mice in different groups were identified for analysis as described in the literature [26]. For tissue preparation and immunohistochemistry, the animals were housed and provided water and a powdered diet until the time of humane sacrifice.

### Microcomputed tomography analysis

The cross-sectional volumetric bone mineral density was measured in right femur diaphysis of the mice in each group after 4 weeks of feeding (for each group, *n* = 6). High-resolution whole-body radiographs of ketamine-anesthetized mice were obtained with the Inveon microcomputed tomography system (Siemens AG, Bensheim, Germany). Using two-dimensional images, a region of interest in secondary spongiosa was manually drawn near the endocortical surface, and cancellous bone morphometric parameters including the bone volume relative to the tissue volume (percentage) and the trabecular thickness (millimeters) were assessed. A trained observer blinded to the composition of the different groups and treatments received analyzed the number and surface area of radiolucent lesions.

### Tissue preparation and immunohistochemistry

Tissue preparation was performed as previously described [29]. The mice were sacrificed after 4 weeks of feeding. The samples were immersed in 4% paraformaldehyde for 24 hours and embedded in 20% sucrose overnight at 4°C. The samples were immersed in O.C.T. compound (Sakura, Tokyo, Japan) for 2 hours at 4°C and cut into 6 μm thick bucco-palatal sections using a cryostat at −20°C. For immunofluorescence staining, the sections were blocked for 30 minutes with 10% normal goat serum and incubated overnight with fluorescein isothiocyanate-conjugated polyclonal rabbit Sca-1 IgG (1:100; Vector Laboratories, Burlingame, CA, USA), 7AAD and Annexin V (Apoptosis Detection Kit; BD Biosciences, Franklin Lakes, NJ, USA) at 4°C. The other sections were stained with hematoxylin and eosin and photographed using a light microscope. In addition, for terminal deoxynucleotidyl transferase-mediated UTP nick-end labeling (TUNEL), an apoptosis detection kit (Millipore Co., Billerica, MA, USA) was used in accordance with the manufacturer’s instructions.

### Cell culture conditions

To determinate the *in vitro* cellular effects of MSCs on MM and 5TGM1 MM cells were co-cultured with MSCs directly or under Transwell® culture conditions. For direct co-culture, MM cells were plated at 5 × 10^5^/ml with or without 5 × 10^5^/ml MSCs for 0, 6, 12 or 24 hours. For in-direct co-culture, Transwell® culture was used. Briefly, the upper chamber (0.5 ml culture medium) contained 0.5 × 10^5^ MSCs, and the bottom chamber (1.5 ml medium) contained either the same number or 5 to 10 times that of MM cells. The Transwell® culture without MSCs in the upper chamber served as the control. In the conditioned culture medium, 0.5 × 10^5^ MSCs were cultured for 3 days. The supernatant was harvested, added to the MM cultures, and then cultured for 0, 6, 12 or 24 hours.

### Flow cytometric analysis

After the MM cells were co-cultured with MSCs for 0, 6, 12 and 24 hours under Transwell® culture conditions, the cells were harvested, washed in PBS and incubated with Annexin-V– fluorescein isothiocyanate and 7AAD-PerCP for 15 minutes at room temperature in the dark. Apoptosis was analyzed on a BD FACSCalibur™ flow cytometer (BD Biosciences, San Jose, CA, USA), as described in the literature [30].

### Western blotting

Total cell lysates for western blots were prepared after lysing cell pellets in radioimmunoprecipitation assay buffer. The lysates were separated through 7.5% SDS-PAGE, transferred to Immobilon™-P nitrocellulose membranes (Millipore, Inc.) and immunoblotted with Fas (1:100) or Fas-L (1:100) antibodies at 4°C overnight. The membranes were subsequently incubated with horseradish peroxidase-conjugated anti-rabbit IgG (1:10,000; Santa Cruz Biosciences, Santa Cruz, CA, USA) for 1 hour, followed by enhancement with a SuperSignal® West Pico Chemiluminescent Substrate (Thermo, Rockford, IL, USA). The bands were detected on BIOMAX MR film (Kodak, Rochester, NY, USA). Each membrane was also stripped using a stripping buffer (Thermo) and reprobed with anti-β-actin antibody to quantify the amount of loaded protein.

### Immunofluorescence staining for multiple myeloma cells or MSCs

MM cells and MSCs co-cultured on dishes were fixed in 4% formaldehyde for 10 minutes. For fluorescence staining, the samples were treated in 0.5% (V/V) Triton X-100 (Novocastra Laboratories Ltd, Newcastle, UK) for 3 minutes and incubated with Annexin V/7AAD. The other cells were blocked with 10% normal goat serum for 30 minutes at 37°C and incubated overnight at 4°C with anti-Fas (1:100; Chemicon International Inc. Temecula, CA, USA) or anti-Fas-L (1:100; Chemicon International Inc.) antibodies. The samples were incubated with tetramethylrhodamine isothiocynate-conjugated or fluorescein isothiocyanate-conjugated secondary antibodies for 2 hours at 37°C. Imaging was performed using an Axiotech Microscope (Carl Zeiss Co. Ltd, Göttingen, Germany).

### Statistical analysis

Data are expressed as mean ± standard deviation for two to four parallel experiments repeated separately. One-way analysis of variance and Fisher’s least-significant difference tests were performed. *P* <0.05 was considered statistically significant. The experiments were performed in triplicate and repeated twice or more to verify the results.

## Results

### Effects of MSC infusion on multiple myeloma model mice

We investigated the 6-week survival rates of mice in each group (for each group, *n* = 14). The effect of MSC administration on the 6-week survival rates of MM model mice is shown in Figure 1B. The survival rate of MM model mice without MSC or L-PAM treatment (MM group) dramatically decreased until all mice died after 6 weeks of feeding. MM model mice with MSC (MSC group) or L-PAM (L-PAM group) administration showed no difference in the survival rate during the 6-week observation period (*P* >0.05). Although the survival rate of mice in the MSC group or the L-PAM group was lower than in the Control group (original nude mice without MM cell injection) (*P* <0.05), the administration of either MSCs or L-PAM achieved prolonged survival compared with the MM group (*P* <0.05). No animal in the negative control group died (the original nude mice without any treatments) after 6 weeks of feeding.

The number and size of cancroid pearls were compared among the four groups (for each group, *n* = 14) after 4 weeks of feeding (Figure 1C). No cancroid pearls were identified in the negative control group (Control group), but significant differences in the number and size of the pearls were observed between the MSC and MM groups (*P* <0.05). Although the number of pearls showed no significant difference, the size of the pearls in the L-PAM group was smaller compared with the MM group (*P* <0.05). These data indicate that MSC administration inhibits both the number and the size of cancroid pearls in MM model mice, while L-PAM administration inhibits only the size of cancroid pearls.

Furthermore, bone resorption in the lumbus and collum femoris of mice in different groups was examined and statistically analyzed through microcomputed tomography after 4 weeks of feeding (for each group, *n* = 3) (Figure 1D). The bone density in both the lumbus and collum femoris of animals in the MM group was much lower compared with the BD in the MSC, L-PAM, or Control groups (*P* < 0.05). Although lower than the Control group (*P* <0.05), no significant differences were observed between the two treated groups (*P* >0.05).

In addition, MM cell metastasis was determined and quantified in the lungs and kidneys of MM model mice after 4 weeks of feeding (for each group, *n* = 5) (Figure 1E). The rate of metastasis in the lung tissue obtained from mice in the MSC group was much lower compared with either the MM or the L-PAM groups (*P* <0.05). Similarly, no significant difference was observed among the MM, MSC, and L-PAM groups in kidney tissue, although the metastasis rate in the MSC group was lowest (*P* <0.05).

### Effects of MSCs on multiple myeloma cells under co-culture conditions

**Figure 2:**
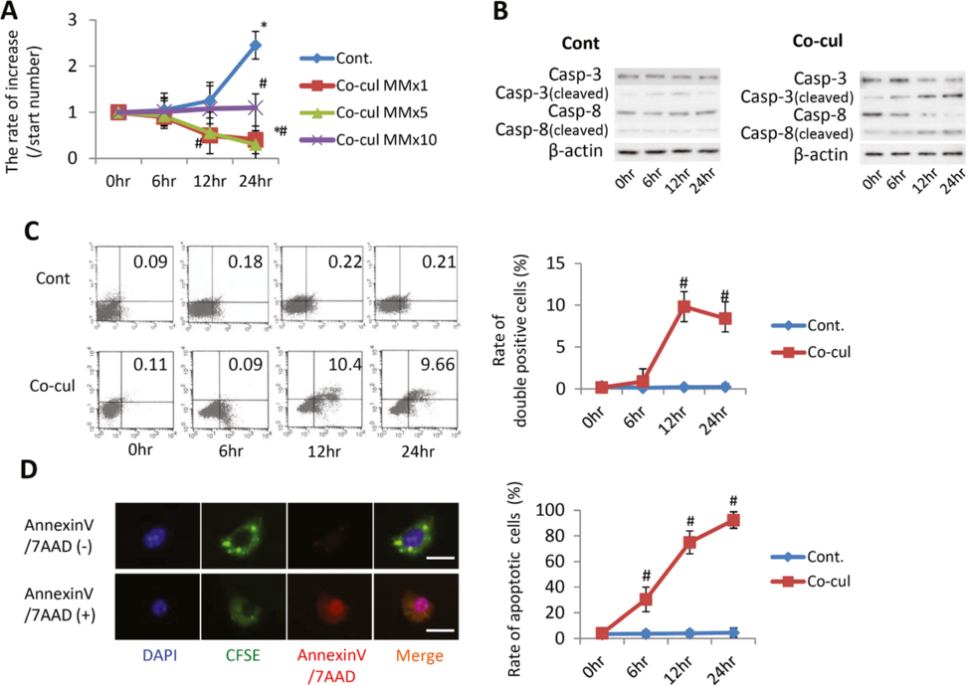
**Relationship between multiple myeloma cells and mesenchymal stem cells under co-culture conditions. (A)** Multiplication of multiple myeloma (MM) cells under co-culture with mesenchymal stem cells (MSCs). **(B)** Western blot analyses of apoptosis markers. Expression levels of cleaved caspase-3 and caspase-8 in MM cells with or without co-culture with MSCs were analyzed through western blotting at 0, 6, 12, and 24 hours (lower table arranged to numerical value from upper data). **(C)** Apoptotic analysis through fluorescence-activated cell sorting (FACS) (*x* axis, Annexin V; *y* axis, 7AAD-positive cells). Apoptosis of MM cells with or without co-culture with MSCs was detected and quantified through FACS at 0, 6, 12, and 24 hours (lower table arranged from upper data). Data presented as mean ± standard deviation (SD) for two parallel experiments. #*P* <0.05 versus the control group (Cont.). **(D)** Rate of apoptotic MM cells under co-culture with MSCs. The microscope pictures represent typical reactions of MM cells in fluorescence staining, where MM cells were prestained for carboxyfluorescein diacetate, succinimidyl ester (CFSE; green) and apoptosis markers using Annexin V and 7AAD (red). Lower table arranged to numerical value from upper data. Data presented as mean ± SD for two parallel experiments. #*P* <0.05 versus the control group (Cont.). DAPI, 4′,6-diamidino-2-phenylindole.

First, we designed three testing co-culture groups, where the number of MM cells was equal to or five to 10 times greater than that of MSCs. MM cells without MSC co-culture served as the control. In the control group, the MM cell number increased 2.5-fold over the initial cell number at 24 hours after co-culture. When the initial number of MM cells was equal to or five times greater than that of MSCs, the inhibitory effect of MSCs on MM cell increase was apparent. However, no significant MM cell increase was observed when the number of MM cells was 10 times the number of MSCs (Figure 2A).

To determine whether the reduction was induced through MM cell apoptosis, caspase-3 and caspase-8 expression in MMs was determined through western blotting. The increased expression of cleaved caspase-3 and caspase-8 was observed in most MM cells after 12 hours of co-culture (Figure 2B). The cleaved caspase-3 and caspase-8 expression in MM cells without co-culture with MSCs was fixed, while the expression of cleaved caspase-3 and caspase-8 in MM cells co-cultured with MSCs was slightly increased. Furthermore, the apoptotic rate of MM cells was examined using fluorescence-activated cell sorting and Annexin V and 7AAD immunofluorescence. We observed that the apoptotic rate of MM cells, determined and quantified through fluorescence-activated cell sorting analysis, was dramatically changed at 12 hours after co-culture with MSCs (*P* <0.05) (Figure 2C). In the case of immunofluorescence, the number of positive apoptotic markers in MM cells significantly increased after 6 hours of co-culture (*P* <0.05) (Figure 2D).

### Influence of Fas/Fas ligand pathway on multiple myeloma cell apoptosis

**Figure 3:**
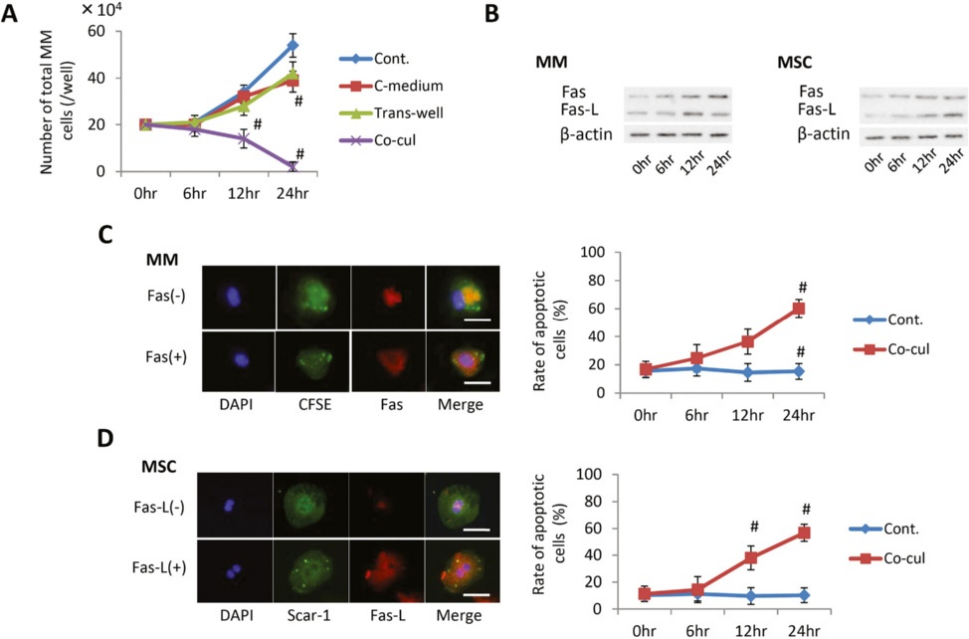
**Fas and Fas ligand analysis in multiple myeloma cells and mesenchymal stem cells under co-culture. (A)** Multiplication of multiple myeloma (MM) cells under different co-culture conditions with mesenchymal stem cells **(**MSCs) (Cont., MM single culture; C-medium, MM culture with conditioned medium from MSC culture; Trans-well, indirect co-culture between MM and MSC; Co-cul, direct culture between MM and MSC). Each data point represents the mean ± standard deviation (SD) of two parallel experiments. #*P* <0.05 versus the control group (Cont.). **(B)** Western blotting analyses of the expression levels of Fas and Fas ligand (Fas-L) in MM cells and MSCs under co-culture condition for 0, 6, 12, and 24 hours. **(C), (D)** The rates of Fas-positive or Fas-L-positive cells were determined through immunofluorescence staining. (C) MM cells were prestained for carboxyfluorescein diacetate, succinimidyl ester (CFSE; green) and then post-stained for Fas (red). (D) MSCs were stained for Scar-1 (green) and subsequently stained for Fas-L (red). Bar = 300 μm. Numbers of Fas-positive MM cells and Fas-L-positive MSCs are shown. Data presented as the mean ± SD for two parallel experiments. #*P* <0.05 versus the control group (Cont.). DAPI, 4′,6-diamidino-2-phenylindole.

Next, we investigated the different conditions of co-culture with MSCs to confirm the necessity of direct contact between MSCs and MM cells for the induction of MM cell apoptosis. Only the direct co-culture group, which shows direct contact between MM cells and MSCs, exhibited a strong inhibitory effect on MM cell growth (*P* <0.05) (Figure 3A). In addition, the expression of Fas in MM cells and Fas-L in MSCs, determined through western blotting, increased at 12 and 24 hours (*P* <0.05) (Figure 3B). The data for immunofluorescence staining showed results similar to those of the western blot analysis (Figure 3C).

**Figure 4:**
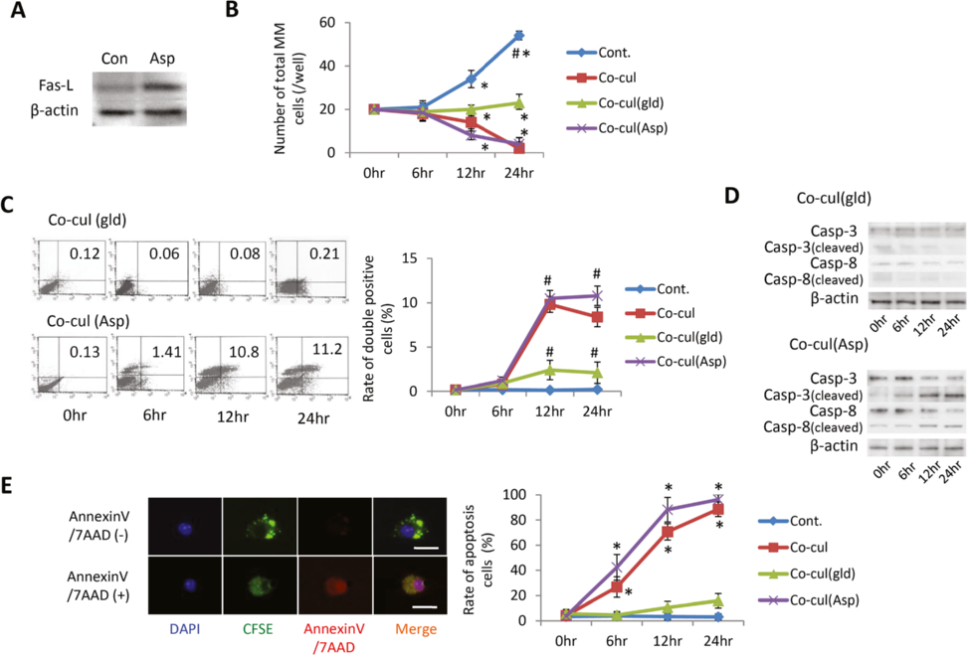
**Effects of Fas ligand levels in mesenchymal stem cells on multiple myeloma cells under co-culture. (A)** Comparison of Fas ligand (Fas-L) expression in mesenchymal stem cells (MSCs) after aspirin treatment. **(B)** Multiplication of multiple myeloma (MM) cells under co-culture condition with MSCs that express different levels of Fas-L. For the graph, the number of MM co-cultured without MSCs (Cont.), with normal MSCs (Co-cul), with MSCs from generalized lymphoproliferative disease mice (Co-cul(gld)) or with MSCs treated with any aspirin (Co-cul(Asp)) were counted after 0, 6, 12 or 24 hours. **(C)** Apoptotic analysis of MM cells under co-culture condition with MSCs expressing different levels of Fas-L (detected and quantified through fluorescence-activated cell sorting (FACS): *x* axis, Annexin V; *y* axis, 7AAD-positive cells). Data presented as mean ± standard deviation (SD) for two parallel experiments. #*P* <0.05 versus Con. **(D)** Expression levels of cleaved caspase-3 in MM cells with or without co-cultured MSCs (expressing different levels of Fas-L) at 0, 6, 12, and 24 hours. **(E)** Rates of apoptotic MM under co-culture with MSCs (expressing different levels of Fas-L). Data presented as mean ± SD for two parallel experiments. #*P* <0.05 versus the control group (Con.). CFSE, carboxyfluorescein diacetate, succinimidyl ester; DAPI, 4′,6-diamidino-2-phenylindole.

MSCs from gld mice, which had no Fas-L, and aspirin-treated MSCs, which had high Fas-L, were used to confirm that the enhancement of Fas-L in MSCs increased the apoptosis of MM cells. Aspirin treatment showed the enhanced expression of Fas-L in MSCs (Figure 4A). A reduction in the number of MM cells co-cultured with aspirin-treated MSCs was less than that in the normal co-cultured group (co-cultured with normal MSCs) at 12 hours (*P* <0.05) (Figure 4B). However, co-culture with the MSCs obtained from gld mice had little influence on MM cell growth. Furthermore, the expression of caspase-3 and caspase-8 in MM cells co-cultured with aspirin-treated MSCs was significantly increased after 12 hours. Under these conditions, the protein expression of cleaved caspase-3 and caspase-8 was reduced after 12 hours. In the case of the gld group, the expression of these proteins remained unchanged (Figure 4C). The rate of apoptotic cell death was further investigated using fluorescence-activated cell sorting (Figure 4D) and immunofluorescence with Annexin V/7AAD (Figure 4E). Aspirin-treated MSCs showed a greater effect on MM cells than normal MSCs (control group) (*P* <0.05), and MSCs obtained from gld mice showed effects similar to those of normal MSCs.

### Effects of MSCs with highly expressed Fas ligand on the multiple myeloma model mice

**Figure 5:**
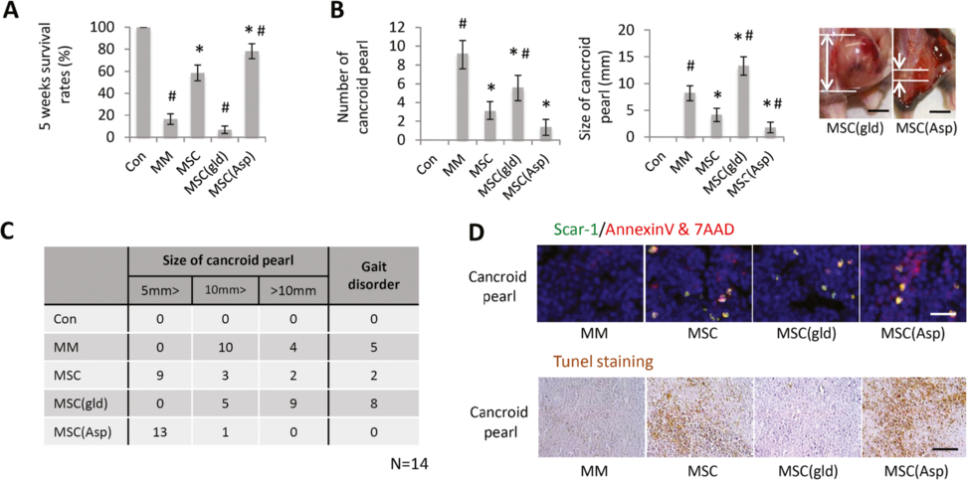
**Effect of mesenchymal stem cells with highly activated Fas ligand on multiple myeloma model mice. (A)** Five-week survival rates of multiple myeloma (MM) model mice. **(B)** Comparison of the number (left) and size (right) of cancroid pearls in MM model mice after treatment with mesenchymal stem cells (MSCs) expressing different levels of Fas ligand (Fas-L). Data presented as mean ± standard deviation (SD) for three parallel experiments. *P <0.05 versus MSC group; #*P* <0.05 versus the control group. Right panel: representative photograph of typical cancroid pearls at the base of the tail (bar = 5 mm). **(C)** Distribution of cancroid pearl size in the four test groups (*n* = 14). **(D)** Apoptosis of MM cells in MM model mice. The pearls in each group were triple-stained for 4′,6-diamidino-2-phenylindole ( blue), Scar-1 (green) and Annexin V/7AAD (red). All groups treated with MSCs had Scar-1-positive cells in the pearls. However, the positive reactions of apoptosis marker in the MSC(gld) group were much lower than those observed in other groups. The MSC(Asp) group had the highest number of reactions among all groups (bar = 5μm). Lower panels stained through the terminal deoxynucleotidyl transferase-mediated UTP nick-end labeling (TUNEL) assay. Positive reactions were observed in both MSC and MSC(Asp) groups. ASP, aspirin; Con, control; gld, generalized lymphoproliferative disease.

The MSCs obtained from normal, gld or aspirin-treated mice were used to examine the 5-week survival rates of the MM model mice. The survival rate in the MM model mice treated with aspirin was much higher compared with normal MSC-treated mice (*P* <0.05). Interestingly, the MSCs obtained from the gld mice showed a reduced survival rate (Figure 5A). Furthermore, the number and the size of cancroid pearls were compared among all groups, and both the number and size in the aspirin-treated group were lower compared with the normal group. However, the gld treated group showed a significantly larger cancroid pearl size compared with the MM model mice without MSC treatment (MM group), although the number of pearls in the MSC gld treated group was less than that in the MM group (Figure 5B). Figure 5C showed the distribution of cancroid pearl size in the MM model mice in each group. Moreover, the presence of MSCs and the apoptosis of MM cells in the pearls of the MM model mice were observed through immunofluorescence and TUNEL staining. The presence of MSCs was positive in all MSC injection groups (Figure 5D).

## Discussion

MM is a malignancy of antibody-secreting plasma cells, where B-cell plasmacytomas stimulate osteoclast activity, and hence bone resorption, resulting in progressive osteolytic lesions [18]. Based on studies concerning the pathogenic role of autoantibodies in MM diseases, recent advances in this field suggest a more central role for B cells in the maintenance of the disease process beyond their roles as precursors for (auto)antibody-producing plasma cells [31]. Particularly, a number of surface molecules and subsequent downstream signaling pathways are involved in the regulation of MM-related bone destroying events, in which bone resorption and formation are no longer balanced as a consequence of the increased activity of osteoclasts, but rather the osteoblast activity is reduced, leading to an uncoupled, or severely imbalanced, bone remodeling process [2]. Clinical data have shown that MM patients with advanced bone lesions might show a reduction of bone formation markers, such as alkaline phosphatase and osteocalcin, together with increased bone resorption markers, such as receptor activator of nuclear factor κB ligand (RANKL) and C-terminal cross-linked telopeptide of type I collagen [32]. Similarly, marked osteoblastopenia and reduced bone formation have also been reported in murine models of MM bone disease [33]. These studies demonstrate that MM cells suppress osteoblast formation and differentiation, and consequently inhibit bone formation.

Recent mounting evidence indicates that MM cells suppress osteoblastogenesis through contact-dependent cell–cell interaction [7,34] and the production of osteoblast-inactivating factors including Wnt inhibitors, such as dickkopf-1 [10] and secreted frizzled-related protein 2 [35], and cytokines, such as CCL3 (also known as macrophage inflammatory protein-1 alpha) [11], hepatocyte growth factor, and IL-3/6 [18]. Osteolytic lesions in MM are only observed adjacent to intramedullary plasma cell foci or plasmacytomas, supporting the idea that MM cells might secrete factors that promote the activation of osteoclasts and the inactivation of osteoblast function to replace bone loss [36]. More effective approaches to cure MM-related bone disease, in addition to the correction of osteoblast function, should therefore be reflected in therapeutic modalities aimed at inducing MM cell death.

Researchers in the stem cell field are working to translate the knowledge gained from stem cell biology and function into therapeutic breakthroughs and applications. It is well known that osteoblasts originate primarily from MSCs and are responsible for bone matrix synthesis through the secretion of collagen, which forms strands called osteoid [37]. Osteoclast activity is regulated through the expression of cytokines, such as receptor activator of RANKL, which activates osteoclast differentiation, and osteoprotegerin, which acts as a decoy receptor and inhibits RANKL [38]. Based on this knowledge, MSC-based cytotherapy has established a novel concept for the treatment of MM-related bone disease [39]. Recently, Li and colleagues demonstrated that both systemic and intrabone cytotherapeutic strategies were effective and clinically applicable for treating MM-related bone disease [15], where weekly systemic injections of MSCs restrained MM disease progression through the ability of MSCs to traffic to myelomatous bone and survive for a short period of time [5]. Intrabone injections of MSCs, however, not only inhibited tumor growth in the bone with active MM but also effectively promoted bone formation during disease, remission and delayed MM relapse. Although this study provides a proof-of-concept for the use of MSC cytotherapy to treat large, unhealed, osteolytic lesions and for the systemic inhibition of MM bone disease, the mechanisms of action by which MSC cytotherapy stimulates bone formation and inhibits MM-induced bone tumor growth, are, however, only partially understood [15]. Whether MSCs inhibit or promote cancer growth has developed into a controversy reflected in concern over the use of MSCs, which exhibit a propensity to home to tumors. Once resident in the tumor microenvironment, these cells support tumor growth and spread [14], although the ability of cultured MSCs to support long-term growth of primary MM cells is often limited and not reproducible [40,41]. Therefore, understanding the *in vivo* milieu in which MSCs either inhibit or enhance MM cell survival and metastasis is crucial both to safely develop MSCs as a therapeutic tool and to advance our understanding of the role of tumor stroma in carcinogenesis [16,17]. Moreover, we still do not have a general consensus of what defines these MSCs; the polarization of MSCs into a proinflammatory or an immunosuppressive phenotype showing reversed effects on tumor growth has been observed [17,42]. Similarly, in our present study, Fas-L^high^ MSCs showed significant inhibition, while Fas-L^null^ MSCs showed promoted MM growth, suggesting that the levels of Fas-L expression in MSCs determine, at least in part, the effect of MSCs on cancer growth.

Recent findings suggest that the overexpression of growth differentiation factor 15 in bone marrow MSCs occurs widely in patients with MM, and tumor microenvironment-derived growth differentiation factor 15 is a key survival and chemoprotective factor for MM cells, indicating that the behavior of MSCs might be principally determined by the surrounding environment [43]. The two side effects of MSCs on MM cells identified from previous studies are therefore basically acceptable. To further clarify the role of healthy MSCs in MM metastasis and apoptosis, we examined whether outside-infused MSCs would have therapeutic effects on MM cells in mice under co-culture conditions. The data obtained in the present study are clearly inconsistent with some reports, which have indicated that murine and human MSCs promote breast and coronal cancer growth and metastasis [44,45]. Interestingly, Ma and colleagues showed that human umbilical cord MSCs significantly inhibited the growth of breast cancer cells *in vitro* and *in vivo*[46]. Furthermore, the ability of cytotherapy through placenta-derived adherent cells to impact bone remodeling and increase bone formation in nonmyelomatous SCID-rab mice has been demonstrated [15]. Intralesional mesenchymal cell cytotherapy also resulted in inhibiting growth of H929 MM cells and primary MM cells categorized through global gene expression as high risk. Moreover, placenta-derived adherent cells had no effect on the subcutaneous growth of H929 MM cells in SCID mice, and did not confer a growth advantage to MM cells co-cultured with placenta-derived adherent cells or supportive MSCs [47]. Recently, adipose-derived MSCs, engineered to express the pro-apoptotic ligand TRAIL (also known as TNFSF10), killed MM cells and migrated towards MM cells *in vitro*[48]. These results, together with data in the present study, support the idea that certain phenotypes of MSCs exhibit inhibitory effects on MM cells, such that the anti-myeloma activity of MSCs can be harnessed or enhanced, for example, via gene-modified approaches.

It has been well recognized that MSC therapy potentially offers novel therapeutic modalities that are translatable for clinical treatment of a large variety of pathological conditions or diseases [49]. This development is also true for clinically managing and combating cancer, as MSCs play a central role in the pathogenesis and progression of tumors [5,50]. MSC administration thus reduces solid tumor growth in mice due to an inhibition of tumor cell proliferation, probably resulting from deep modifications of the tumor angiogenesis, regardless of the tumor model and mode of MSC injection [51]. Clinically, current evidence suggests that cytotherapy markedly increases the proportion of MSCs in bone of MM patients, at least for a short period of time [5,15]. As deduced from *in vitro* studies, during this short time, the injected MSCs probably interact with endogenous osteoblast precursors and secrete factors that induce their differentiation into bone-building osteoblasts, while simultaneously directly interactions with osteoclast precursors to secrete factors that attenuate the formation of bone-resorbing osteoclasts [15]. Notably, similar to osteoblasts, MSCs might produce a high level of decorin protein, which inhibits osteoclast formation and promotes osteoblast differentiation [8]. Following the identification of the potential for MSCs to enhance engraftment of hematopoietic stem cells, increase osteoblast activity and suppress osteoclast activity [52], MSCs recruited hematopoietic elements that inhibit inflammatory conditions typically associated with MM growth in bone [15]. Along with recent findings in this field [17,42], it is speculated that MM progression is restrained, directly and indirectly, through anti-inflammatory factors produced by the injected MSCs or endogenous cells recruited to myelomatous bone after cytotherapy. The findings that MSCs express high levels of anti-inflammatory and antineoplastic factors, such as SERPINF1 and decorin, support this concept [5]. Decorin also attenuates MM cell growth [5]. Although certain soluble factors produced by MSCs might mediate part of their therapeutic activities, cytotherapy at a remote site (subcutaneous) was found to have no effect on MM bone disease or growth [15], suggesting that MSCs must be present in bone marrow to elicit their antimyeloma effects. Indeed, only MSCs injected directly into bone might efficiently induce an antimyeloma environment. Systemically injected MSCs significantly promote bone formation or restrain MM growth because relatively few of those MSCs can transmigrate and traffic to bone [5]. Recent results, however, also suggest that MSCs might be attracted to bone through MM cells or conditions induced through MM or melphalan treatment. More importantly, MSCs might be cleared in various tissues, but exhibit higher survival rates in the implanted bone or lymph nodes and therefore could be detected in these tissues at 2 to 3 days after intravenous or intracardiac injections, respectively [5]. The accumulation of MSCs in lymph nodes, however, might partially explain their immunomodulatory properties. In fact, evidence suggests that intravenously injected MSCs might localize in the lymph nodes of experimental mouse models of autoimmunity [53,54]. This body of work might also explain the fewer numbers and smaller sizes of cancroid pearls in the neck and root tail of the MSC-treated MM mice in the present study.

Recent studies have revealed that exogenously injected MSCs were not detectable *in vivo* for long periods of time; the majority of these cells disappeared within 3 to 5 weeks [15,55]. Clinically, this phenomenon might be advantageous because it limits the duration of the intervention, and these observations support the notion that most of their activities are mediated through the touch-and-go mechanisms of bystander cells, although proof of such evanescence is thus far not well defined [5,56]. In support of using allogeneic MSCs for MM, Li and colleagues recently demonstrated that intralesionally injected human placenta mesenchymal cells exert similar therapeutic effects in SCID-rab mice [15]. Together, these studies raise an intriguing possibility: if we could understand how MSCs induce MM cell death, then perhaps we could exogenously manipulate MSCs to effectively manage MM and saved a large number of lives. An important, yet unelucidated, question raised by our study is whether a majority of MSCs transmigrate to the myelomatous bone to kill MM cells after intravenous injection, or traffic to lymph nodes to exert inhibitory effects on MM cells via the secretion of anti-inflammatory factors.

The potential role of molecules involved in altered B-cell longevity, particularly those involved in apoptosis (for example, Fas/Fas-L modulators), and those that might alter activation thresholds of B cells in the development of autoimmunity, might contribute to the clinical management of MM [27,31]. Unfortunately, however, we still know relatively little about this issue. Recently, a number of studies have reported the effects of the Fas/Fas-L pathway on fluoride-induced or melatonin-induced cell apoptosis [57,58]. In the present study, it was shown that MSCs counterattack MM cells using the same mechanism as observed in other cancer cells; namely, Fas-mediated apoptosis. Fas and Fas-L are co-expressed on primary MSCs that might kill co-cultured MM cells. Co-expression of Fas and Fas-L by MSCs calls into question the functionality of the extrinsic apoptosis pathway in these cells. Although Mazar and colleagues detected Fas expression on MSCs, stimulation of Fas with different concentrations of anti-Fas antibody did not result in any apoptotic response [23]. At this stage, we were not able to identify the exact molecular mechanism of Fas-mediated pathway inactivation in MSCs, but we might narrow this process to events between Fas protein trimerization and caspase-8 activation. As previously demonstrated, caspase-8 deficiency resulted in the inhibition of apoptosis of Jurkat cells, and blocking with Fas Fc protein prevented bone marrow MSC-induced apoptosis in 80% [23]. Other studies have shown that the transformation of the intracellular domain of Fas protein expressed in human MSCs prevents the trimerization of the receptor and blocks the activation of apoptotic pathway activation [59]. MM cells might thus be susceptible to the induction of apoptosis through MSCs.

Briefly, we showed that MSCs act directly on MM cells, inhibiting proliferation. MSC-induced apoptosis in MM cells is evidenced by an increase in the Annexin/7AAD-positive cell population. Most of this effect can be attributed to the Fas/Fas-L pathway. These results confirm and extend previous reports [60,61]. In our MM model, the activation of both caspase-3 and caspase-8 was observed, suggesting that two main pathways of procaspase activation – the intrinsic mitochondrial pathway and the extrinsic death receptor pathway – are both involved in MSC-induced apoptosis of MM. Having determined the effects of MSCs activated through aspirin (with highly activated Fas-L) in the MM model mice, these MSCs resulted in a more effective clinical outcome compared with MSCs from the gld mice. We thus expected that MSCs with high Fas-L expression would be extremely effective in inhibiting MM growth and metastasis. These results are consistent in principle with those of another study, showing that infused MSCs moved immediately to the tumor site [62]. However, the positive TUNEL reactions within MSCs from the gld mice were much lower than the others, suggesting that MSCs without Fas-L have no capacity to kill MM cells.

## Conclusion

Fas-L is expressed on MSCs to induce MM cell apoptosis under co-culture conditions. Furthermore, Fas-L activated through aspirin effectively inhibits the growth and metastasis of MMs *in vitro* and in *vivo*. Our results showed the additive or synergistic anti-MM activity of MSCs with highly activated Fas-L, measured on the basis of cell growth, apoptosis, and modest survival improvement of MM-bearing mice, indicating that the levels of Fas-L expression in MSCs determine, at least partially, the effect of MSCs on cancer growth. Our finding has opened up a new way of thinking about the effect of MSCs against MM cells and a new way of moving forward. Further research should aim at improving the trafficking of infused MSCs to myelomatous bone, clarifying and increasing the anti-inflammatory therapeutic potential of these cells during active disease or maintenance therapy compared with the typical administration of drugs, such as L-PAM.

## Abbreviations

Fas-L: Fas ligand; gld: Generalized lymphoproliferative disease; IL: Interleukin; L-PAM: Lymphocyte Peyer’s patch adhesion molecules; MM: Multiple myeloma; MSC: Mesenchymal stromal cell; PBS: Phosphate-buffered saline; RANKL: Receptor activator of nuclear factor κB ligand; TNF: Tumor necrosis factor; TUNEL: Terminal deoxynucleotidyl transferase-mediated UTP nick-end labeling.

## Competing interests

The authors declare that they have no competing interests.

## Authors’ contributions

IA, SL, YM and KA were involved in the practical achievement of these experiments. IA, CC, YA and F-MC collected, analyzed and interpreted the data. SS designed the study and provided financial and administrative support. IA, SL, SS and F-MC wrote the manuscript. SL, CC, SS and F-MC revised the manuscript critically for important intellectual content. All authors read and approved the manuscript for publication. Each author participated sufficiently in the work to take public responsibility for appropriate portions of the content.

## Authors’ information

IA and SL are co-first authors. SS and F-MC are co-last authors.
